# Evolutionary origin and functioning of pregenital abdominal outgrowths in a viviparous insect, *Arixenia esau*

**DOI:** 10.1038/s41598-019-52568-w

**Published:** 2019-11-06

**Authors:** Waclaw Tworzydlo, Mariusz K. Jaglarz, Laura Pardyak, Barbara Bilinska, Szczepan M. Bilinski

**Affiliations:** 1 Department of Developmental Biology and Invertebrate Morphology, Institute of Zoology and Biomedical Research, Faculty of Biology, Jagiellonian University in Krakow, Gronostajowa 9, 30-387 Krakow, Poland; 2 Department of Endocrinology, Institute of Zoology and Biomedical Research, Faculty of Biology, Jagiellonian University in Krakow, Gronostajowa 9, 30-387 Krakow, Poland

**Keywords:** Morphogenesis, Entomology, Embryology

## Abstract

Although pregenital abdominal outgrowths occur only rarely in pterygote insects, they are interesting from the evolutionary viewpoint because of their potential homology to wings. Our previous studies of early development of an epizoic dermapteran, *Arixenia esau* revealed that abdominal segments of the advanced embryos and larvae, growing inside a mother’s uterus, are equipped with paired serial outgrowths. Here, we focus on the origin and functioning of these outgrowths. We demonstrate that they bud from the lateral parts of the abdominal nota, persist till the end of intrauterine development, and remain in contact with the uterus wall. We also show that the bundles of muscle fibers associated with the abdominal outgrowths may facilitate flow of the haemolymph from the outgrowths’ lumen to the larval body cavity. Following completion of the intrauterine development, abdominal outgrowths are shed together with the larval cuticle during the first molt after the larva birth. Using immunohistochemical and biochemical approaches, we demonstrate that the *Arixenia* abdominal outgrowths represent an evolutionary novelty, presumably related to intrauterine development, and suggest that they are not related to serial wing homologs.

## Introduction

Two basic reproductive strategies, i.e. the oviparity and viviparity can be found in various vertebrate and invertebrate lineages^1–4^. Oviparous species deposit eggs comprising spheres/platelets of proteinaceous yolk, lipid droplets and glycogen particles (i.e. reserve materials) that are used during embryogenesis. In viviparous species, the embryos are retained in a female reproductive system or the body cavity. In these cases, females deposit larvae that are ready to start active life and feed. A wide spectrum of nutritional strategies have been found in viviparous taxa. On one side of this spectrum, the embryos developing inside the mother’s reproductive system use substances accumulated (during vitellogenesis) in large yolky oocytes; this mode is termed the lecithotrophy. On the other side, the embryos are fed with nutritional molecules transferred from the tissues of the mother. This mode is termed the matrotrophy. Interestingly, in certain invertebrate species both nutritional modes are combined to a different degree (for further details see^2,3^).

Recently, we have been intensively studying different aspects of matrotrophic viviparity in the epizoic dermapteran, *Arixenia esau* (Arixeniidae)^5–8^. We have shown that the embryogenesis of this species can be divided into two clearly recognizable phases that undergo in disparate compartments of the reproductive system^5,7^. Initially, the embryos develop inside the terminal ovarian follicles and use reserve materials (lipid droplets and yolk spheres) stored during oogenesis in relatively large oocytes^7,9^. After formation of the germ band, the embryos are relocated to the transformed lateral oviducts (termed collectively the uterus) where they develop till the offspring birth. Detailed SEM studies revealed additionally that the second (i.e. intrauterine) phase of *Arixenia* development comprises of three stages: early embryos (before dorsal closure, enveloped by a chorion and two extraembryonic cellular layers or “membranes”: the amnion and serosa), late embryos (after dorsal closure, and still encompassed by the chorion, amnion and serosa) and the first instar larvae (after hatching from the chorion). Early and late embryos develop freely in the fluid filling the uterus, whereas the first instar larvae come into direct contact with the uterus wall (see^7^ for further details). Our analyses have also revealed that the initial stage of the intrauterine phase (as the whole intraovarian one) relies on the lecithotrophic mode of nourishment. After the onset of the second intrauterine stage and till the birth of the offspring, the embryos/larvae rely, nearly exclusively on the matrotrophic nourishment mode^7,8^. We have shown, therefore, that in *Arixenia* the dorsal closure coincides with an important physiological modification: a shift from the lecitothrophic to matrotrophic nourishment. Finally, our studies have demonstrated that abdominal segments of *Arixenia* embryos and larvae are furnished with paired multilobed outgrowths^7^. After hatching (that is liberation from the chorion) the outgrowths adhere to the uterus epithelium. This leads to the formation of a series of small contact sites between the mother and embryo tissues that collectively constitute a “scattered” placenta-like organ. It has been suggested, in the preceding papers, that this organ might be responsible for the transfer of maternally derived nutrients and oxygen to the developing embryos^7,8,10^.

In principle, abdomens of adult winged insects (Pterygota) are devoid of non-sexual appendages. The only two exceptions to this rule are the appendages on the fourth abdominal segment of male sepsid flies^11^ and lateral abdominal sensory and secretory organs (LASSOs) of Southeast-Asian hemipteran taxon Bennini^12^. In contrast, abdomens of immature stages (larvae and even pupae) of pterygotes are often equipped with pregenital abdominal appendages/outgrowths, e.g. nymphal gills of mayflies, tracheal gills of whirligig beetle and megalopteran larvae, denticular outgrowths (“gin trap devices”) of *Tenebrio molitor* pupae. These structures do not serve sexual functions, i.e. they are either respiratory or protective. The origin as well as homology of pregenital abdominal outgrowths of winged insects have been analyzed and discussed in several papers^13–16^. It transpires from these analyses that the abdominal appendages/outgrowths might represent either serial wing homologs (see^14,16,17^ for information on the origin and function of wing homologs) or *de novo* formed morphologically complicated extensions of the abdominal segments^11,12^.

The aim of the analyses presented in this paper was to gain insight into the origin as well as functioning of the serial abdominal outgrowths of *Arixenia* embryos and larvae. We show that the outgrowths bud from the lateral parts of abdominal nota (terga) of early embryos and remain attached to these exoskeletal elements till the end of the intrauterine development. They degenerate and become shed together with the cuticle of the 1^st^ instar larva during the first larval molt after the birth. These observations, together with immunohistochemical and biochemical data imply that *Arixenia* abdominal outgrowths represent *de novo* formed protrusions of the dorsal exoskeletal plates (sclerites) and are not evolutionarily related to (serial) wing homologs and/or their elements. We show additionally that the outgrowths contain bunches of muscle fibers, attached to the epithelium lining the outgrowths’ wall. We suggest that coordinated contractions of these fibers are responsible for the flow (pumping) of the haemocelic fluid from the outgrowths’ lumen to the larval haemocel.

## Results and Discussion

### The origin of the abdominal outgrowths

**Figure 1:**
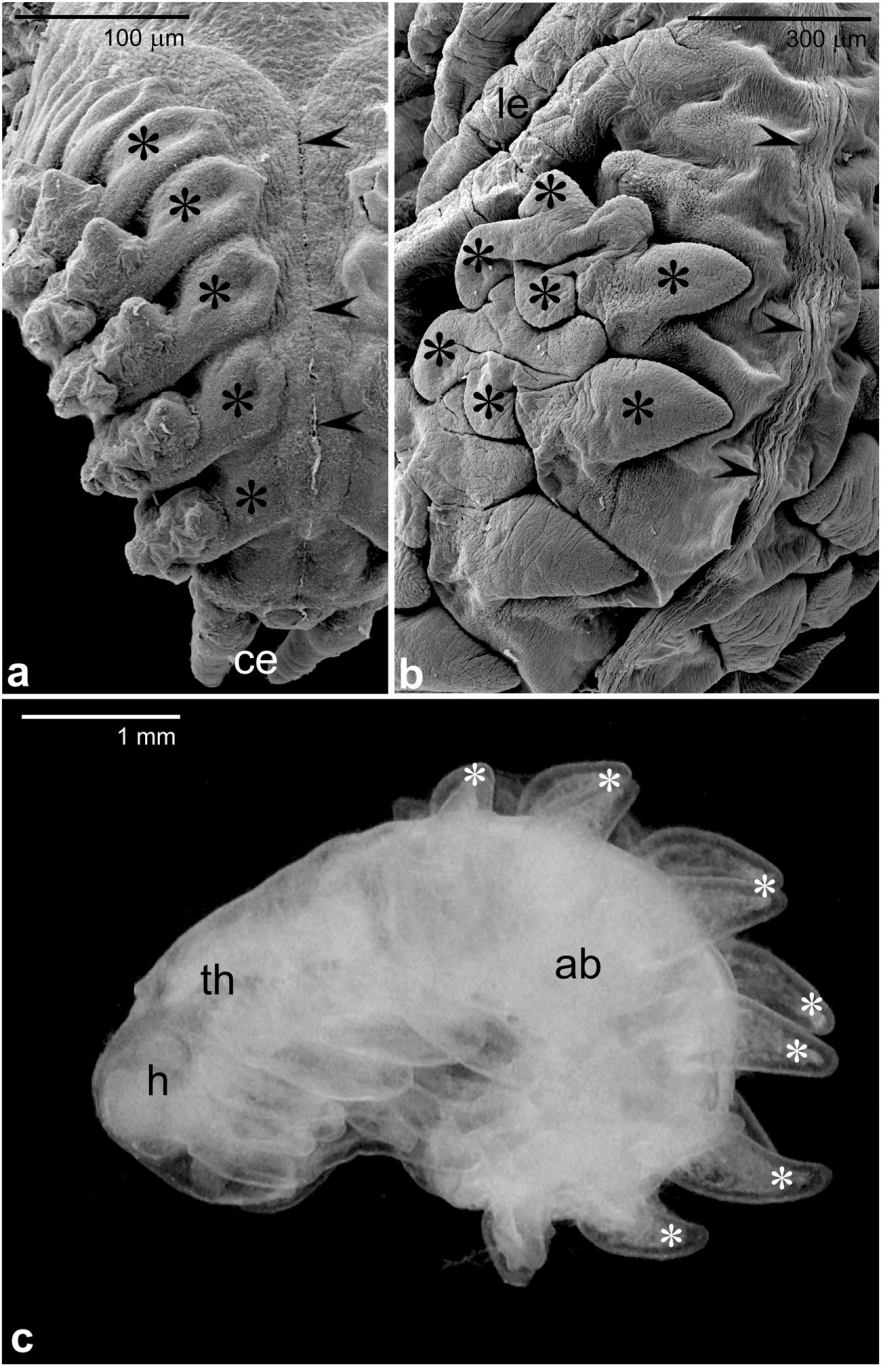
Pregenital abdominal outgrowth development. **(a,b)** Fragments of embryo abdomens during the dorsal closure. Arrowheads point to the region where epithelial flanks meet and fuse during the dorsal closure. Asterisks indicate abdominal outgrowths (**a**) or their lobes (**b**); Cerci (ce), legs (le). **(c)** Lateral view of the first instar larva after liberation from the egg envelope. Head (h), thorax (th), abdomen (ab). Note that lobes of the abdominal outgrowths (asterisks) are arisen and protrude almost perpendicularly from the abdomen. (**a**,**b**) SEM, (**c**) stereomicroscope.

Till the end of the second intraovarian stage of embryogenesis, i.e. before the “hatching”, the *Arixenia* embryos develop surrounded by a chorion, amnion and serosa^5^ (Fig. S1). As these layers are thin and do not adhere directly to the embryonic cuticle, the manual “dissection” of embryos is relatively easy. To find out which part(s) of the abdominal segment participate(s) in the formation of the outgrowths we examined such dissected embryos fixed before and after the dorsal closure. Our analyses revealed that the outgrowths arise as paired slightly elongated buds attached to the dorsal side of the first eight abdominal segments, next to the region where ectodermal flanks meet and fuse as the dorsal closure advances (Fig. 1a). As embryos develop, the outgrowths remain attached to the dorsal side of abdominal segments (Fig. 1a,b); simultaneously they grow and ramify into lobes (Fig. 1a,b). Consequently, the first eight abdominal segments of late stage 2 embryos are partially covered with flattened ramified outgrowths (Fig. 1b) that adhere (cling) tightly to the dorso-lateral aspects of the embryo. At the onset of the last intrauterine developmental stage, the outgrowths “straighten out” (Fig. 1c) and come into contact with the uterus epithelium, forming a dispersed (scattered) placenta-like organ (see^7^ for further details).

**Figure 2:**
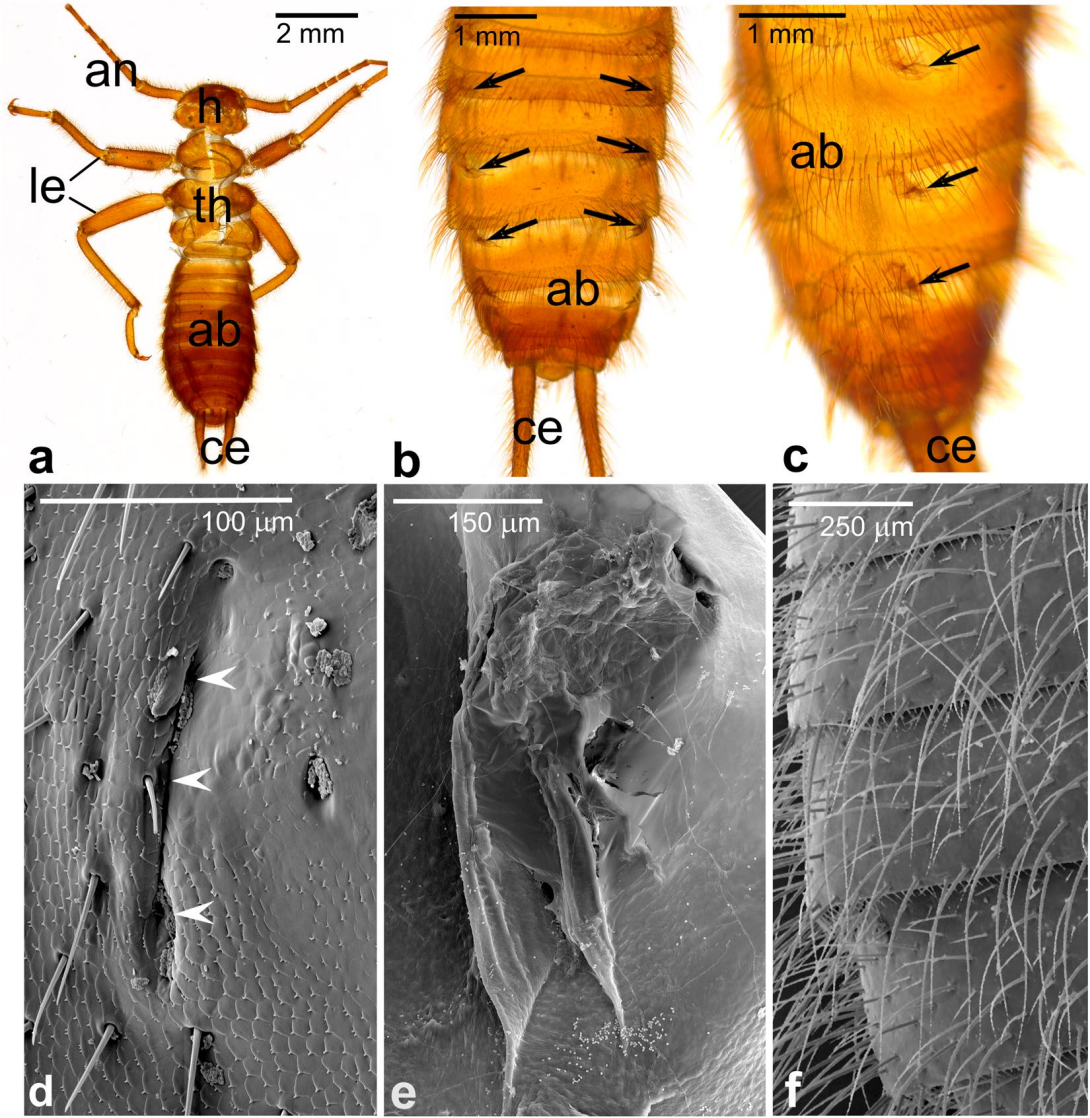
Pregenital abdominal outgrowth fate. **(a–e)** Larval exuvies shed during the 1^st^ molt after birth. Antennae (an), head (h), thorax (th), abdomen (ab), cerci (ce). Only two pairs of legs (le) were retained after the molt. Note that in addition to bristles, the abdomens bear collapsed serial outgrowths (arrows in **b**,**c**) located on lateral sides of abdominal nota (terga). **(d)** Collapsed, folded inwards, outgrowth (white arrowheads). (**e)** Inner surface of collapsed outgrowth with small pieces of decomposed tissue attached. **(f)** Abdominal nota of older larvae are flat, devoid of outgrowths or their remnants and bear elongated bristles only. **(a–c)** stereomicroscope; **(d–f)** SEM.

Next, we analyzed the fate of the abdominal outgrowths after the termination of the intrauterine development. For this aim we collected and examined larval exuvies (Fig. 2a–e). We found that abdomens of the smallest exuvies (shed during the 1^st^ molt after the birth) bear, in addition to bristles, collapsed and apparently not functional serial outgrowths (Fig. 2a–e). The latter are located on lateral sides of the first eight abdominal nota (terga). The analysis of the exuvies in the SEM shows that the collapsed outgrowths consist almost solely of folded inwards “shrunk” cuticle (Fig. 2d,e). Only small pieces of unidentified, apparently decomposed, tissue were found attached to the inner cuticle surface (Fig. 2e). The abdominal nota of older larvae are flat and bear elongated bristles only (Fig. 2f). Altogether, our findings indicate that the outgrowths, likely involved in matrotrophic nourishment of the progeny, degenerate and are eliminated together with the cuticle shed during the 1^st^ molt after the birth of the first instar larva.

**Figure 3:**
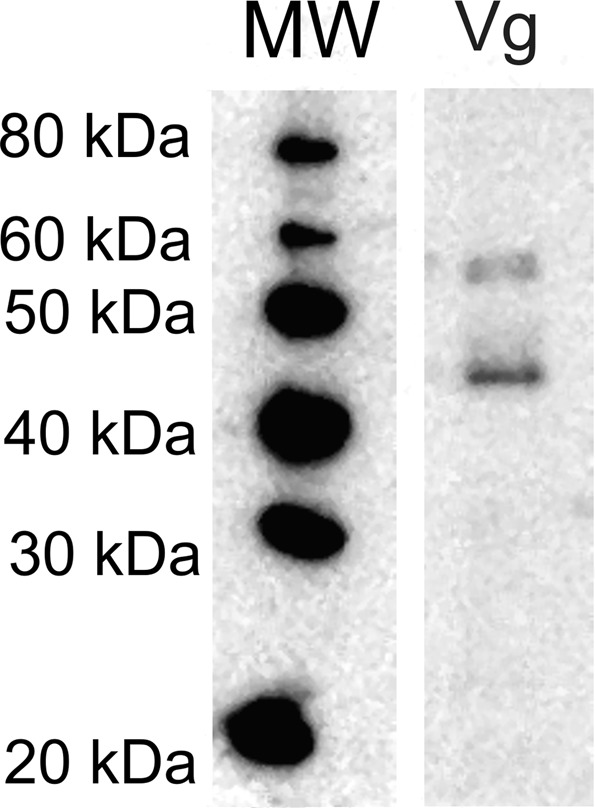
Detection of Vestigial (Vg) in *Arixenia* embryo tissues. Immunoblots with antibodies against Vg; MW – molecular weight markers.

Vestigial (Vg) is a key factor regulating specification of dorsal structures (wings and halterae) during *Drosophila* development^18–20^. To explore the possibility that *Arixenia* abdominal outgrowths may represent serial wing homologs, we asked whether the ortholog of *vestigial* (*vg*) is expressed in these structures. First, we tested whether Vg homologue is present in homogenates of the *Arixenia* embryos and larvae. Western blot analysis with the antibody against *Drosophila* Vg revealed a protein with molecular weight around 46 kDa (Fig. 3), which well corresponds with a predicated molecular weight of Vg^18^. Next, we used the same antibody for immunofluorescence labeling of paraplast sections. These experiments showed that Vg is not expressed in the tissues of embryonic outgrowths (Fig. S2a), while positive immuno signal was detected in nerve cord cells (Fig. S2b).

The above results indicate that abdominal outgrowths of *Arixenia* embryos/larvae represent morphologically complicated protrusions of the abdominal nota (terga). This notion is supported by the following observations:

1. The outgrowths bud from the dorsal side of the embryo.
2. They are attached to the abdominal nota till the end of the intrauterine phase of development. Even collapsed nonfunctional outgrowths remain attached to the nota of the exuvies shed during the 1^st^ molt after the birth.
3. Although a homolog of *Drosophila* Vg is apparently present in homogenates of whole embryos, the tissues of the embryonic outgrowths do not express this protein.

### Functioning of the outgrowths

**Figure 4:**
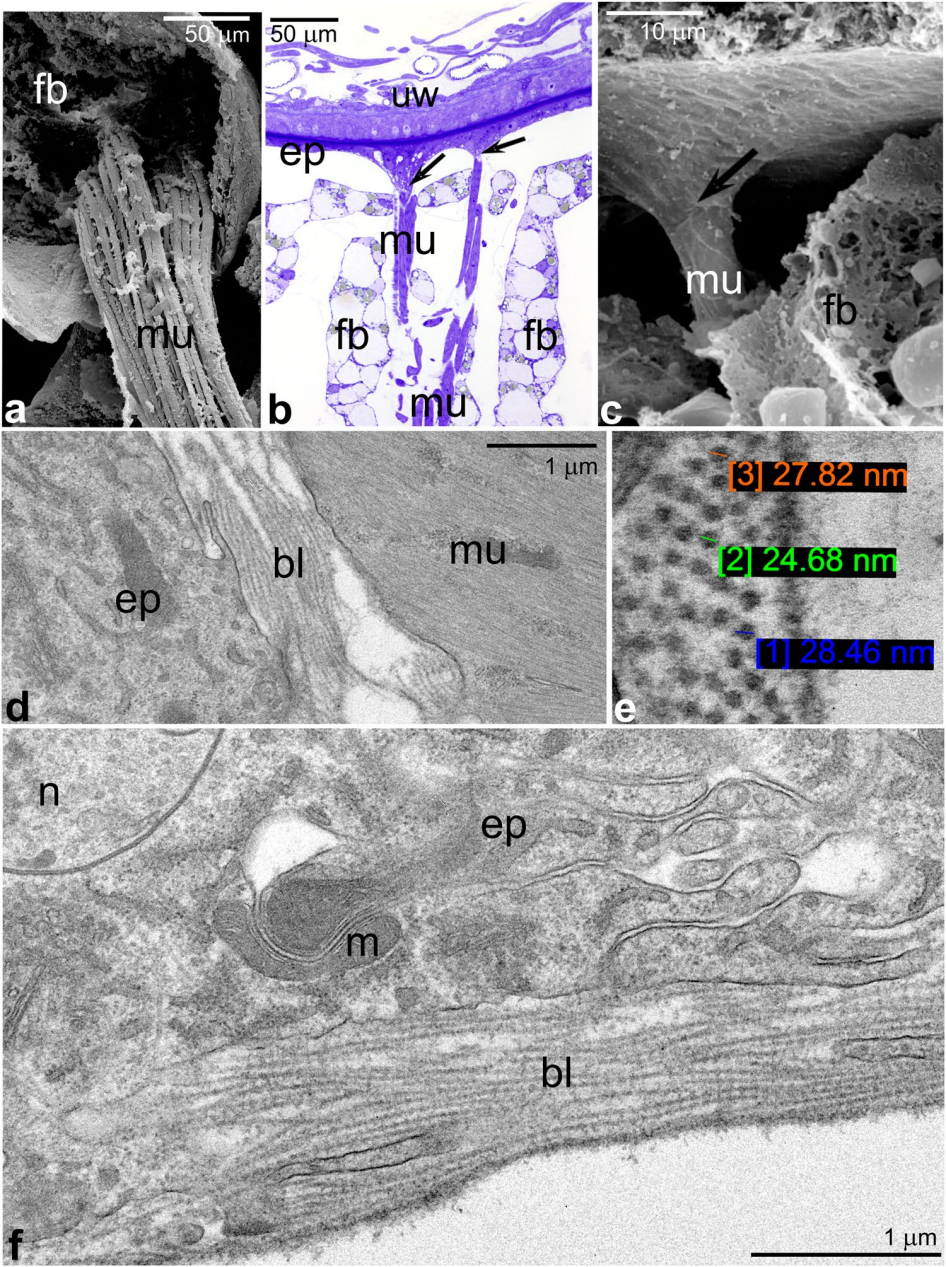
Structure of pregenital abdominal outgrowth. **(a–c)** The lumen of the outgrowth. Note muscle fibers (mu) surrounded by fat body stripes (fb). Uterus wall (uw), arrows point to the muscle attachments. **(d–f**) Basal part of epithelial cells (ep), basal lamina (bl). Note that the muscle fibers (mu) are attached to filamentous basal lamina (bl). Mitochondrion (m), epithelial cell nucleus (n). Filaments of the basal lamina are parallel arranged with characteristic regular axial periodicity. Cross-sectioned filaments are shown and measured in **(e). (a,c)** SEM, **(b)** LM, **(d–f)** TEM.

**Figure 5:**
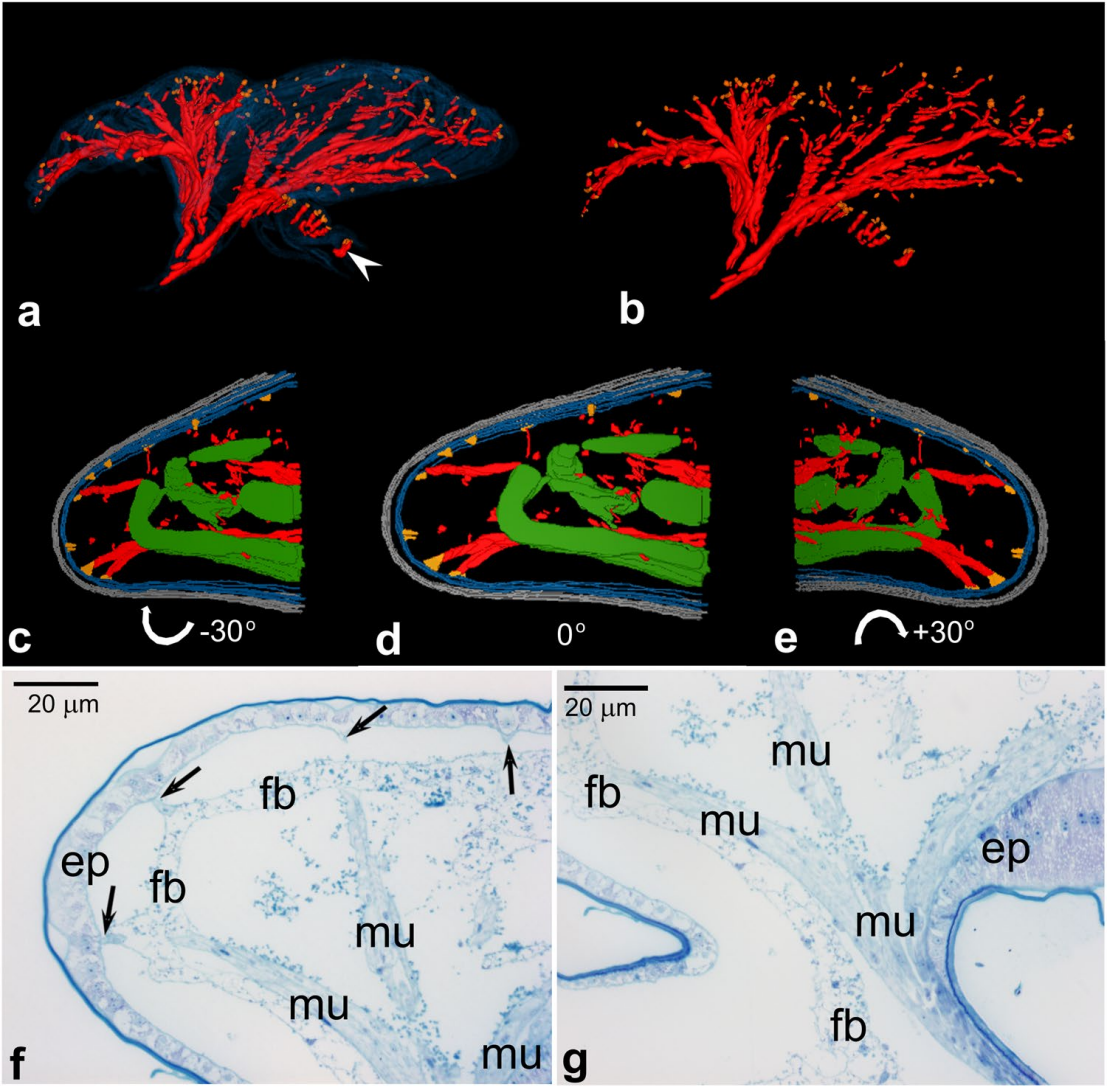
Organization of pregenital abdominal outgrowth. **(a,b)** Computer aided 3D reconstruction of the abdominal outgrowth. For these reconstructions 120 serial sections were used. Muscle fibers (red), muscle attachments (orange), epithelial cells (blue, omitted in **b**), arrowheads indicate muscles attached to the cuticle surrounding the outgrowth base. **(c–e)** Partial 3D reconstructions of the outgrowth “empty” lobe. For these reconstructions 10 serial sections were used. Muscle fibres (red), muscle attachments (orange), epithelial cells (blue), cuticle (gray), fat body (green), white arrows and corresponding numbers indicate rotation angle. **(f,g)** Fragments of the representative sections used for the reconstructions shown in. (**a,b**) Epithelial cells (ep), fat body (fb), muscle fibers (mu). Arrows point to the muscle attachments. (**f**–**g**) LM.

Our previous studies^7^ have shown that *Arixenia* serial abdominal outgrowths are subdivided into four morphologically disparate lobes. One of them is almost “empty” comprising a spacious lumen lined with flat epithelial cells (Fig. 4b). The remaining three are more “solid” and lined with a monolayer of highly prismatic epithelial cells that fill the lobe lumen almost completely. Interestingly, the lumen of both lobe types is integrated with the haemocoel of the embryo. In addition to the epithelial cells, the outgrowth’s lobes comprise several bunches of thin muscle fibers and elongated stripes of the fat body (Fig. 4a–c). The muscle fibers are not directly attached to the cuticle covering the lobes but adhere to the basal lamina associated with the basal parts of the epithelial cells (Fig. 4b–d). The basal lamina consists of thick (27 nm in diameter, on average), parallel arranged filaments with characteristic regular axial periodicity of roughly 30 nm (Fig. 4d–f). Additional muscle fibers are attached to the basal lamina of the epithelial cells, next to the base of the outgrowths (Fig. 5a, white arrowhead).

As the information that can be extracted from incidental (and not properly orientated) sections is insufficient for the reconstruction of the whole organ, we performed a computer aided 3D reconstruction of serial semi-thin histocryl/epon sections through fully grown larval outgrowths. These reconstructions visualized exact relationships between constituents (e.g. muscle fibers, their attachments) of the outgrowths. From 10 to 120 semithin (1–2 µm thick sections) were used in our reconstructions (Fig. 5a–e; animated reconstructions are presented in Videos S1 and S2). Analysis of the serial sections and obtained 3D images revealed that bunches of muscle fibers that enter the outgrowth base, split into individual fibers penetrating to all the lobes of the outgrowth (Fig. 5a–g). Interestingly, the muscle attachments are not distributed uniformly but are preferentially gathered along the external surface of the outgrowth’s lobes (Fig. 5c–f).

To approximate efficiency of the outgrowths functioning, we estimated the volume of the haemolymph that might be exchanged between the larval haemocoel and the lumen of the open outgrowths lobes during the contraction of the muscle fibers. Preliminary assumptions for these calculations were as follows: (1) the muscle fibers present in all 16 lobes of a given larva contract simultaneously; (2) during contraction, the muscle fibers shorten on average 10%^21^; (3) 25 or 50% of the larval haemocoel is filled with organs, i.e. the rest is filled with the haemocoelic fluid (two possibilities). Our calculations showed that during every (simultaneous) muscle contraction the volume of the haemolymph pumped out from the outgrowths equals 7 to 10% of the total volume of the haemolymph present in the larval body cavity. It transpires from these approximations that all abdominal outgrowths of a given specimen, or collectively its placenta-like organ, represent an efficient and effective system responsible for the pumping of the haemolymph and its constituents towards larval tissues. For additional information on nutrition and respiration of *Arixenia* embryos/larvae see^7,8,10^.

## Conclusions

The results of the present paper suggest that the serial abdominal outgrowths of *Arixenia* are not homologous to canonical dorsal appendages (wings and/or halterae in dipterans) but represent an interesting evolutionary novelty presumably related to the matrotrophic mode of embryo nourishment. Thus, the *Arixenia* outgrowths extend the short list of the abdominal pregenital structures (as abdominal appendages of sepsid flies^11^ and LASSOs^12^) that had evolved independently (*de novo*) and are not related to any of the thoracic appendages. However, an alternative interpretation of our results, based on the current hypothesis of dual origin of insect wings (i.e. from dorsally located paranotal lobes and ventrally located proximal leg segments; for review see^16,22–24^), cannot be excluded. In the light of this hypothesis, the *Arixenia* abdominal outgrowths may be considered as structures homologous solely to the dorsal (paranotal) part of the wing primordia. This interpretation would correspond with serial arrangement (distribution) of the *Arixenia* abdominal outgrowths. Regardless of the exact nature of the outgrowths on abdominal segments of *Arixenia* larvae, their existence reveals high potential and plasticity of the seemingly conserved (ca. 350 million years old) insect bauplan.

## Materials and Methods

### Animals

The larvae, adult females and exuvies of *Arixenia esau* Jordan 1909 were collected in small caves, inhabited by colonies of *Cheiromeles torquatus* bats, in Bintulu District area, Sarawak, Malaysia in February 2010. In this study, 10 gravid females, about 20 embryos, and 15 first instar larvae were used; at least three specimens were examined in each experiment. The female reproductive system, embryos and first instar larvae were dissected and fixed either in 4% formaldehyde or in 2.5% glutaraldehyde in 0.1 M phosphate buffer (pH 7.3). The exuvies, as well as several first instar larvae dissected from the uteri and fixed, were placed in the same buffer and photographed in a Nikon SMZ 1500 stereomicroscope (Nikon, Tokyo, Japan).

### Ethical approval

All applicable national and institutional guidelines for the animal use were followed. No live vertebrates and/or higher invertebrates were used in this study.

### Light and transmission electron microscopy

Dissected material was rinsed and postfixed in a mixture of 1% osmium tetroxide and 0.8% potassium ferrocyanide for 30 min at 4 °C, dehydrated in the series of ethanol and acetone and embedded either in Glycid Ether 100 (formerly known as Epon 812) (Serva, Heidelberg, Germany) or Histocryl (Agar Scientific Ltd., Stansed, Essex, UK). Semi-thin sections (0.7–1 μm thick) were stained with 1% methylene blue and examined in a Leica DMR microscope (Heidelberg, Germany). Ultrathin sections (80 nm thick) were contrasted with uranyl acetate and lead citrate according to standard protocols and analyzed in a Jeol JEM 2100 transmission electron microscope (TEM) at 80 kV.

### Scanning electron microscopy (SEM)

Samples for SEM analyses, were fixed and postfixed as described above, then dehydrated in graded series of ethanol, critical-point dried, coated with gold and examined in a Hitachi S-4700 scanning electron microscope at 25 kV (for further details see^6^).

### Western blot analysis

To analyze Vestigial homologue protein expression, embryos dissected from the uteri were homogenized and sonicated in a cold Tris/EDTA buffer (50 mM Tris, 1 mM EDTA, pH 7.5), supplemented with a broad-spectrum protease inhibitors (Sigma-Aldrich), as described previously by Hejmej *et al*.^25^. The protein concentration was estimated by the Lowry dye-binding with bovine serum albumin (BSA) as a standard (Bio-Rad Labs, GmbH, München, Germany). Thereafter, 40 μg of protein was solubilized in sample buffer (Bio–Rad Laboratories) and heated at 99.9 °C for 5 min. After denaturation, proteins were separated by sodium dodecyl sulphate–polyacrylamide gel electrophoresis (SDS–PAGE) on 10% polyacrylamide gels under reducing conditions. Separated proteins were transferred onto a polyvinylidene difluoride membranes using a wet blotter. Non-specific binding sites were blocked with a solution of 5% (wt/v) non-fat dry milk containing 0.1% (v/v) Tween 20, and the membrane was incubated with a rabbit polyclonal antibody against Vestigial (1:500; the antibody was raised against the *Drosophila* Vestigial and was kindly provided by Prof. S.B. Carroll, University of Wisconsin-Madison, USA) at 4 °C overnight. Next, the membranes were washed briefly with TBST and incubated in a goat anti-rabbit IgG secondary antibody conjugated to horseradish peroxidase (1:3000; Vector Laboratories, Burlingame, CA, USA) for 1 h at room temperature. Immunoreactive protein was detected by chemiluminescence with the western blotting luminol reagent as described previously by Mruk and Cheng^26^ and images were captured with a ChemiDocTM XRS + System (Bio-Rad Laboratories). The molecular weight of the target protein was estimated by reference to standard proteins (MagicMark™ XP Western Protein Standard, Thermo Scientific).

### Immunolocalization of Vestigial

For the immunohistochemical analyses the material was fixed in 4% formaldehyde. Samples were dehydrated in series of ethanol and HistoChoice^®^ Clearing Agent (Sigma-Aldrich) and embedded in paraplast. The blocks were cut into 1 to 5-μm-thick sections. Slide-mounted sections were dewaxed in HistoChoice^®^ Clearing Agent (Sigma-Aldrich), rehydrated gradually through a series of ethanol dilutions and rinsed in water (for further details see^10^). Blocking of non-specific binding sites was performed with casein blocking buffer (Thermo Fisher) overnight at 4 °C prior to the incubation with the rabbit anti-Vestigial antibody diluted 1:500. In parallel performed control experiments, the primary antibody was omitted. After overnight incubation at 4 °C in humid chamber, Cy3 goat anti-rabbit secondary antibodies (Life Technologies) were used. Incubation with secondary antibodies was carried out for 4 h at room temperature. After rinsing with PBS, the sections were mounted in ProLong Gold antifade reagents with DAPI (Invitrogen) and analyzed in the DMR Leica epifluorescence microscope (FM) equipped with appropriate filters.

### 3D reconstruction of muscle fibers distribution in abdominal outgrowths

To reconstruct spatial distribution of muscle fibers and their attachment points, we serially sectioned four abdominal outgrowths of the first instar larva. The sections (1–2 μm thick) were photographed in a DMR Leica microscope. Resulting micrographs were aligned to form virtual stacks and required structures were contoured using TrakEM2 plugin of the ImageJ software^27^. 3D reconstructions were created using 3D viewer and Z-projections plugins of the same program^27^.

## Supplementary information

Supplementary Information

Video S1

Video S2

## Data Availability

The datasets generated during and/or analyzed during the current study are available from the corresponding author on reasonable request.
