# Supplementary material for: Evolutionary origin and functioning of pregenital abdominal outgrowths in a viviparous insect, Arixenia esau
Source: Sci Rep. 2019 Nov 6;9:16090. doi: 10.1038/s41598-019-52568-w (PMC6834671; doi:10.1038/s41598-019-52568-w)
Supplement: Supplementary file 1 — Supplementary Information [file 41598_2019_52568_MOESM1_ESM.pdf]

Supplementary Information

Evolutionary origin and functioning of pregenital abdominal outgrowths in a viviparous insect, Arixenia esau

Waclaw Tworzydlo, Mariusz K. Jaglarz, Laura Pardyak, Barbara Bilinska and Szczepan M. Bilinski

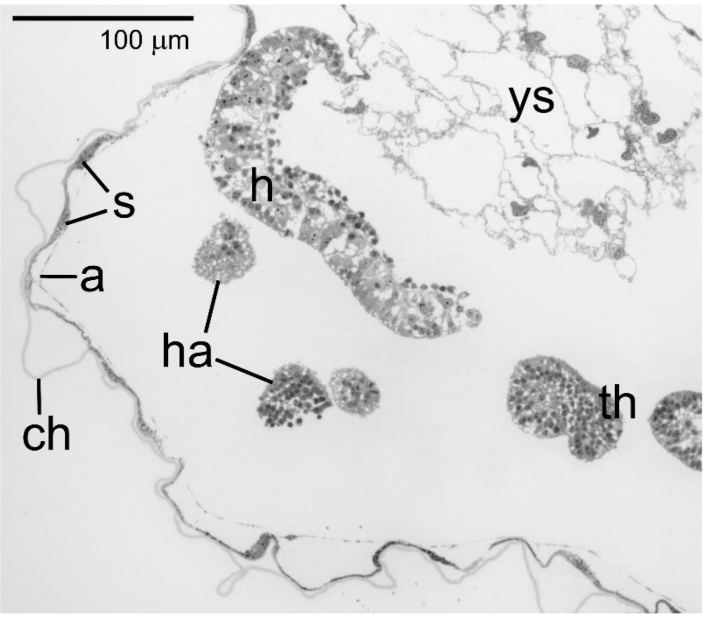

Figure S1. Fragment of the early (1st intrauterine stage) *Arixenia* embryo. Note that during this stage the embryo is surrounded by acellular chorion (ch) and two extraembryonic cellular membranes: amnion (a) and serosa (s). Head (h), head appendages (ha), thorax (th), yolk sac (ys). LM.

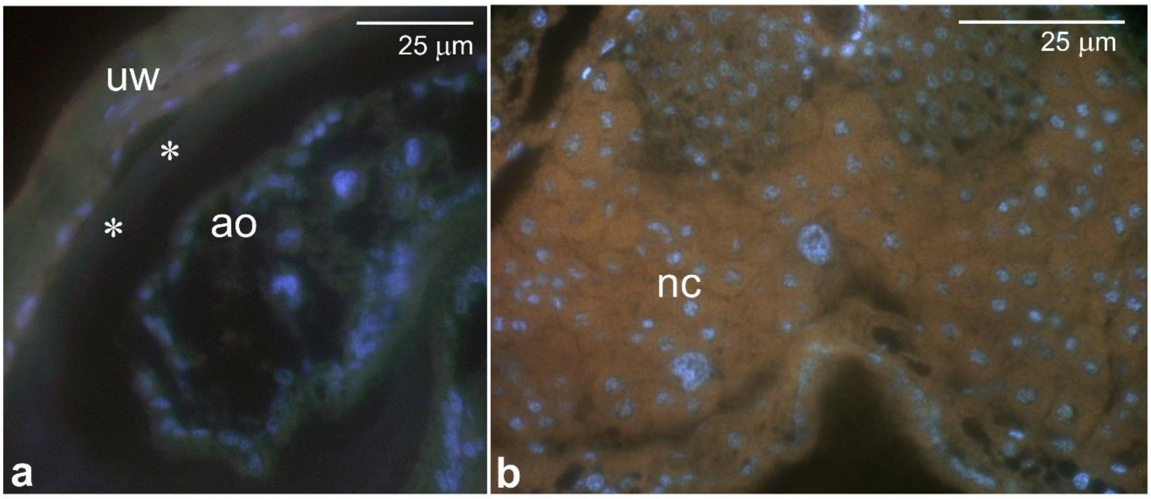

Figure S2. Immunolocalization of Vg protein in selected tissues of more advanced embryos; nuclei stained with DAPI. (**a**) Dorsal abdominal outgrowth (ao) surrounded by chorion (asterisks) and uterus wall (uw). Note that the outgrowth is Vg-negative. (**b**). Vg-positive nerve cord (nc). (**a**-**b**) FM.

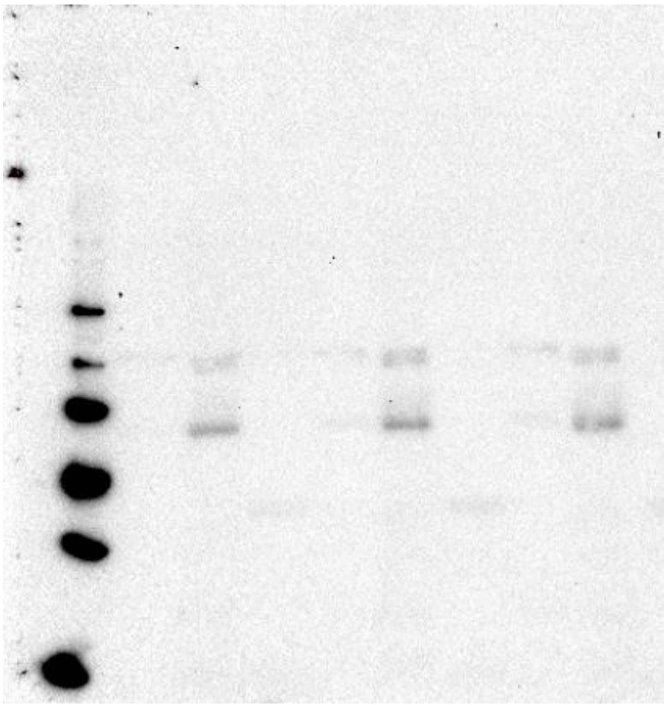

Figure S3 The full-length gel presented in Fig. 3.

Video S1. Animated 3D reconstruction of abdominal outgrowths.

Video S2. Animated 3D reconstruction of abdominal outgrowths.
